# Cobb Angle Measurement of Spine from X-Ray Images Using Convolutional Neural Network

**DOI:** 10.1155/2019/6357171

**Published:** 2019-02-19

**Authors:** Ming-Huwi Horng, Chan-Pang Kuok, Min-Jun Fu, Chii-Jen Lin, Yung-Nien Sun

**Affiliations:** ^1^Department of Computer Science and Information Engineering, National Pingtung University, Pingtung, Taiwan; ^2^Department of Computer Science and Information Engineering, National Cheng Kung University, Tainan, Taiwan; ^3^Institute of Medical Informatics, National Cheng Kung University, Tainan, Taiwan; ^4^Department of Orthopedics, National Cheng Kung University Hospital, Tainan, Taiwan

## Abstract

Scoliosis is a common spinal condition where the spine curves to the side and thus deforms the spine. Curvature estimation provides a powerful index to evaluate the deformation severity of scoliosis. In current clinical diagnosis, the standard curvature estimation method for assessing the curvature quantitatively is done by measuring the Cobb angle, which is the angle between two lines, drawn perpendicular to the upper endplate of the uppermost vertebra involved and the lower endplate of the lowest vertebra involved. However, manual measurement of spine curvature requires considerable time and effort, along with associated problems such as interobserver and intraobserver variations. In this article, we propose an automatic system for measuring spine curvature using the anterior-posterior (AP) view spinal X-ray images. Due to the characteristic of AP view images, we first reduced the image size and then used horizontal and vertical intensity projection histograms to define the region of interest of the spine which is then cropped for sequential processing. Next, the boundaries of the spine, the central spinal curve line, and the spine foreground are detected by using intensity and gradient information of the region of interest, and a progressive thresholding approach is then employed to detect the locations of the vertebrae. In order to reduce the influences of inconsistent intensity distribution of vertebrae in the spine AP image, we applied the deep learning convolutional neural network (CNN) approaches which include the U-Net, the Dense U-Net, and Residual U-Net, to segment the vertebrae. Finally, the segmentation results of the vertebrae are reconstructed into a complete segmented spine image, and the spine curvature is calculated based on the Cobb angle criterion. In the experiments, we showed the results for spine segmentation and spine curvature; the results were then compared to manual measurements by specialists. The segmentation results of the Residual U-Net were superior to the other two convolutional neural networks. The one-way ANOVA test also demonstrated that the three measurements including the manual records of two different physicians and our proposed measured record were not significantly different in terms of spine curvature measurement. Looking forward, the proposed system can be applied in clinical diagnosis to assist doctors for a better understanding of scoliosis severity and for clinical treatments.

## 1. Introduction

**Figure 1:**
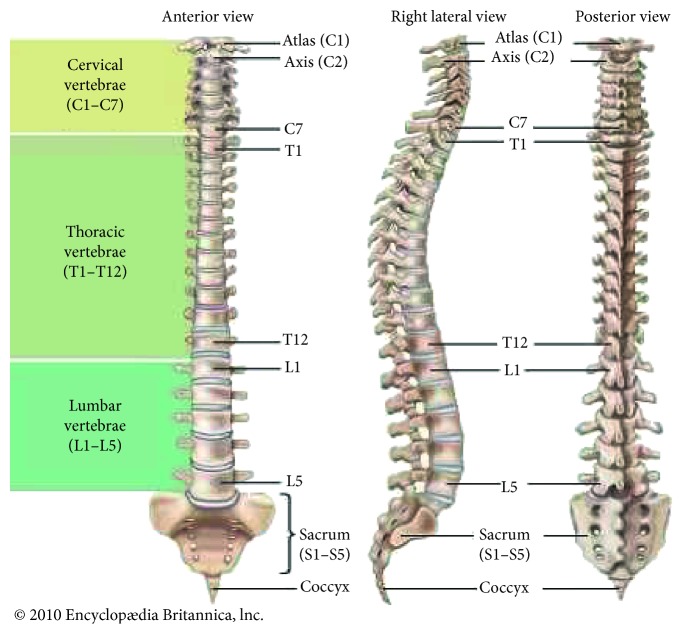
The vertebral column [1].

The spine is one of the most important parts of the human body. It provides a human with many significant functions, for example, carrying the weight of the body and protecting the spinal cord and nerves within. As shown in Figure 1, the spine consists of 33 vertebrae that are subdivided into five regions: cervical (C1–C7), thoracic (T1–T12), lumbar (L1–L5), sacrum (S1-S5), and coccyx (Co1–Co4). The upper 24 vertebrae are separated and movable providing the spinal column with flexibility. The lower 9 vertebrae are fixed, and 5 sacral vertebrae are fused to form the sacrum and 4 coccygeal vertebrae are usually fused to form the coccyx after adolescence [1].

**Figure 2:**
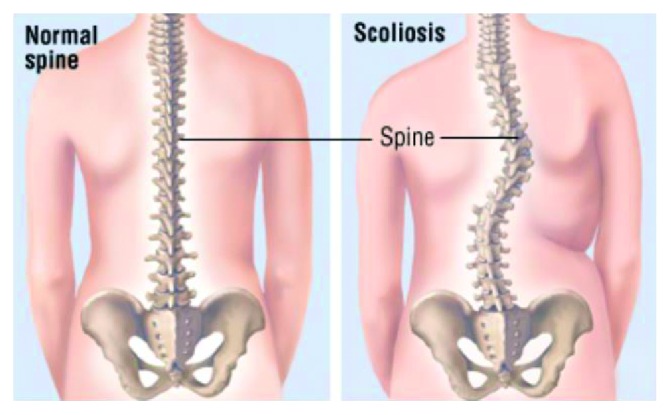
Normal spine and scoliosis [2].

A normal spine should be straight and centered over the pelvis when viewed from the front and viewed from the back. Scoliosis is a condition where the spine abnormally curves towards the left or the right side and when the sideways curve of the spine is greater than 10 degrees. A person's spine with scoliosis will look like a C- or S-shaped curve as shown in Figure 2.

**Figure 3:**
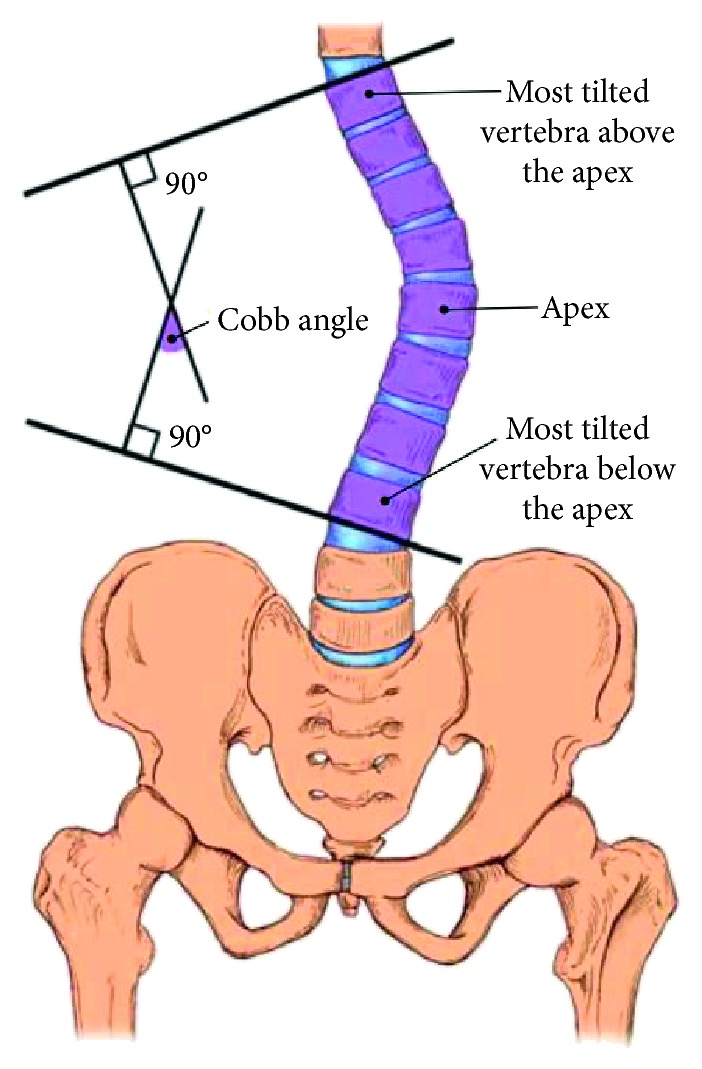
Measurement using the Cobb method [3].

Symptoms associated with scoliosis may include pain in the back or shoulders, osteoarthritis, and even respiratory or cardiac problems in severe cases. In order to establish a diagnosis of scoliosis, a physician measures the degree of spinal curvature on imaging scans such as X-rays, CT scans, and MRIs. The most common quantification of scoliosis is the Cobb angle [4], which was originally proposed by the American orthopedic surgeon John Robert Cobb. The Cobb angle was formally adopted as the standard quantification of scoliosis by the Scoliosis Research Society (SRS), founded in 1966. The measurement of Cobb angle involves estimating the angle between the two tangents of the upper and lower endplates of the upper and lower end vertebra, respectively, as shown in Figure 3. The severity definition for scoliosis is determined using the Cobb angle as shown in Table 1. The condition of a spine is associated with the spinal curve instead of scoliosis when the Cobb angle is less than 10 degrees. A Cobb angle in the range of 10 to 20 degrees is considered as mild scoliosis. Scoliosis severity is moderate when the Cobb angle ranges from 20 to 40 degrees. A Cobb angle greater than 40 degrees denotes severe scoliosis.

The current widely adopted standard for scoliosis diagnosis and treatment decisions is the manual measurement of Cobb angles, which refers to the internal curvature of the spine trunk. Despite the fact that manual measurement has been working for the last decade, it is difficult for clinicians to make accurate measurements because of the large anatomical variation of patients from different age group and the low tissue contrast of X-ray spinal image. This usually results in a large number of interobserver or intraobserver errors. Therefore, the development of automated computer measurement is an important research topic to provide a reliable and robust quantitative assessment of scoliosis.

In the literature, there are many articles that deal with interesting relevant topics. Giannoglou and Stylianidis [6] provided a review article about the Cobb angle calculation and image-based modelling techniques for measurement of spinal deformities. In this article, Cobb angle measurement includes X-ray image processing that attempts to detect the locations of vertebrae in order to calculate the Cobb angle of each AP view X-ray spinal image. In general, the sequences of the image processing involve the following stages: (a) image acquisition, (b) corner detection of the vertebra, and (c) a final stage for global estimation of the spine curvature.

Moura et al. [7] proposed a set of techniques to (1) isolating the spine by removing other bone structures, (2) detecting vertebrae locations along the spine using the progressive threshold method, and (3) detecting vertebrae lateral boundaries. The author used a tree data structure to prune redundant information and to merge regions that were too small. The detected vertebrae boundaries were used to measure the Cobb angle of spine curvature. Okashi et al. [8] proposed a fully automatic solution for spine segmentation and curvature quantification from X-ray images of mice. Their approach consists of three stages: preparation of the region of interest, spine segmentation, and spine curvature quantification. The stage for preprocessing the region of interest involves three operations: (a) aligning the mouse skeleton, (b) cropping of ROI, and (c) denoising and enhancing the cropped ROI. The spine segmentation stage first uses the Otsu method to obtain the initial segmentation and then further refines it. The refinement first applies two grayscale morphology operations tophat and topbot, to reduce noise and maximize contrast. Next, the spine border is refined by using a complex iterative process to determine a high intensity value for modifying border pixels. Finally, polynomial fitting methods are applied to refine edges of the spine. Two different indexes, SRM_1_ and ARM_2_, are proposed to measure the spine curvature. This method had some shortcomings: (a) it requires complex image processing techniques to segment the spine and (b) it does not separate each vertebra that cannot compute the most useful measure, which is the Cobb angle.

Mukherjee et al. [9] selected the best filter of the four denoising techniques: bilateral filters [10], nonlocal means filters [11], principal neighborhood dictionaries nonlocal means filtering [12], and block matching three-dimensional filtering [13]. Due to the poor contrast of radiographs, histogram equalization was applied to enhance image contrast, and the Otsu thresholding method was used to find the Canny edge points of vertebrae. Finally, a Hough transform [14] was used to detect the two straight lines of the upper endplate of the uppermost vertebra involved and the lower endplate of the lowest vertebra involved. The two detected lines were then used to find the Cobb angles for comparison. Lecron et al. [15] proposed a learning method that combines scale-invariant feature transform (SHIF) local descriptors [16] with a multiclass SVM to detect vertebra anterior corners. However, these methods require complicated image processing stages that involve image filtering, enhancement, segmentation, and feature extraction to obtain vertebra assessment, which make the techniques computationally expensive and temptable to errors caused by the variations in X-ray spinal images.

Recently, deep convolutional neural networks (CNNs) have demonstrated enormous potential in the field of medical image analysis [17, 18]. Unlike traditional machine learning methods, deep neural networks do not require any handcrafted features for training and can be trained end-to-end for object detection and semantic segmentation. As such, a CNN network is a suitable choice for extracting the vertebral regions of a spine. In biomedical image segmentation, recent successes in precise image segmentation were achieved by using a U-Net architecture [19]. In the U-Net, contextual information is propagated to upsampling layers by concatenating the output of lower layers to high layers, providing more feature channels. Al Arif et al. [20] applied the U-Net and shape-aware U-Net to segment the cervical vertebrae. The authors modified the crop and copy operation into the concatenation operation that obtained an average Dice similarity coefficient (DSC) of 0.9438 for U-Net and 0.944 for shape-aware U-Net. The authors also compared with other methods such as ASM-G [21], ASM-M [22], and ASM-RF [23]. Their DSCs are 0.774, 0.877, and 0.883. These results reveal that the performance of our proposed work is very close to the one in [24] and should be better than the abovementioned methods [21–23]. Additionally, the modifications of a U-Net such as Residual U-Net [24] and Dense U-Net architecture [25] were also applied to segment the thoracic and lumbar vertebra for comparison.

**Figure 4:**
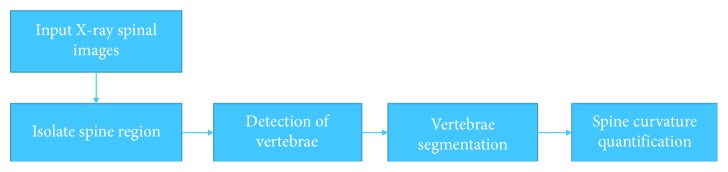
Flow chart of the proposed system.

In this paper, we proposed an automatic system for the measurement of spine curvature from X-ray images. A flow chart of the proposed system is shown in Figure 4. The proposed system includes four stages: isolation of the spine region, detection of vertebrae, segmentation of vertebrae, and spine curvature quantification. The isolation of the spine region stage starts from the image preprocessing procedure that includes resizing an input image and cropping the region of interest (ROI) of a spine. Afterwards, image processing techniques are applied to detect the locations of vertebrae using a progressive threshold method. And then we apply a convolutional neural network (CNN) to segment vertebrae. Unlike the work of Moura et al. [7], we used an analogous voting mechanism to separate each vertebra. The final stage is to compute the spinal curvature by applying the criterion of Cobb angle measurements.

The rest of the paper is organized as follows.  Section 2 introduces the proposed methods and data material for the experiments. The experimental results and discussion of the proposed system are in Section 3. Finally, Section 4 presents the conclusion and future works.

## 2. Materials and Methods

### 2.1. Experimental Materials

**Figure 5:**
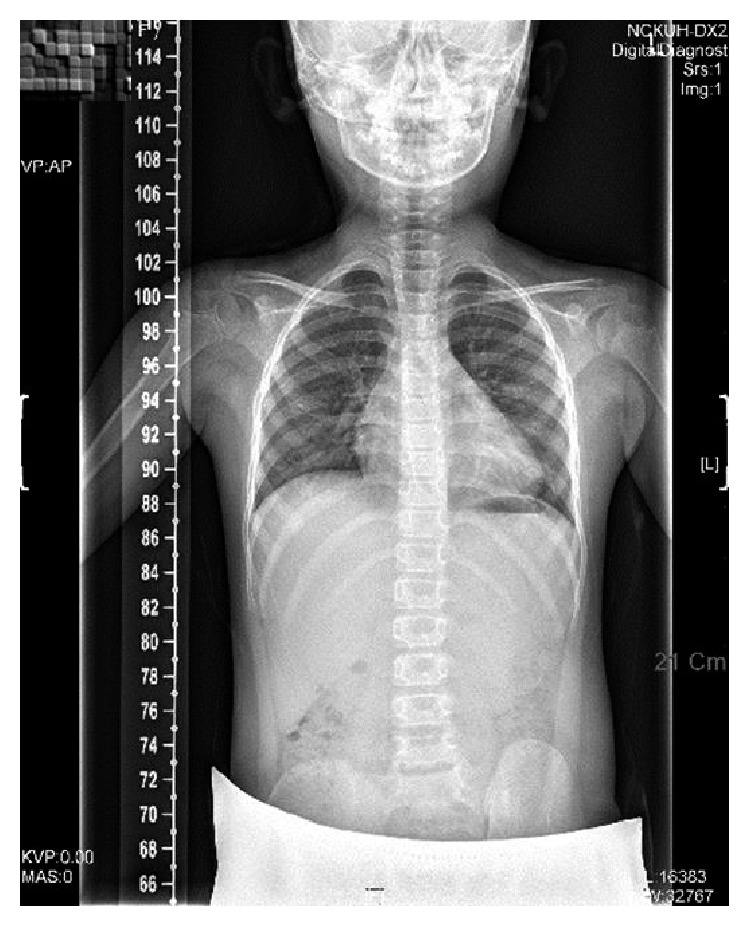
2D X-ray spinal image in the AP view.

The X-ray spinal images used in the experiments were acquired from the National Cheng Kung University Hospital using EOS medical imaging system (EOS company, Paris). Prior to experiments, all participants were informed about the study's aims and procedures, which include the removal of identification for privacy protection and signed consent forms approved by the Institutional Review Board of National Cheng Kung University Hospital (IRB number: A-ER-105-013). The images are 2D X-ray spinal images in the anterior-posterior view (AP view) in grayscale format as shown in Figure 5 with a size of width: 1056 to 3028 pixels and height: 1996 to 5750 pixels. In total, thirty-five images captured from young scoliosis subjects were used in this study, each depicting a complete spine which includes 12 thoracic and 5 lumbar vertebrae for the following segmentation process. Most of the X-ray spinal images are about 3000 × 5000 pixels in size.

### 2.2. Proposed Methods

#### 2.2.1. Isolating the Spine Region

**Figure 6:**
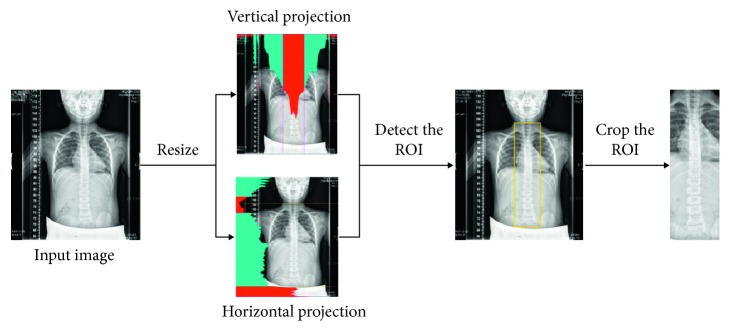
Spine region isolation.

The isolation of the spine region stage is applied to decide upon the region of the interest (ROI) of the spine. In order to make the processing more efficient, we first reduce the size of all spinal AP view images to a quarter of its original size. In this stage, we focused on the region between the thoracic and the lumbar vertebrae (i.e., from T1 to L5 vertebrae) in the X-ray AP view spinal images. The region is defined as the spine region of interest (spine ROI). Figure 5 shows the image columns with the brighter pixels indicating columns where the spine is located. Therefore, we first vertically align the large structures including the head, spine, and hips and then compute the intensity histogram of a vertical projection. We select columns that are between the mean intensity plus or minus one standard deviation as the left and right boundaries of ROI as shown in Figure 6. Another interesting observation from Figure 5 is that the intensity of the spine near the thoracic vertebrae is relatively low, but the spine regions of the lumber vertebrae appear brighter. As a result, we used the intensity histogram of a horizontal projection to detect the lowest extrema as the upper boundary of ROI and the position of the largest discontinuous position as the lower boundary as shown in Figure 6. The detected spine ROI is then cropped for sequential spine detection and segmentation.

#### 2.2.2. Vertebrae Detection

After extracting the spine region, we further detect the locations of vertebrae from the spine ROI image. In general, the spine usually appears with a higher intensity in the cropped spine ROI; therefore, we can detect the edges of the spine by using the sums of the intensity and gradient. There are three steps for detecting the vertebrae: (1) detection of the central line segment (CLS), (2) detection of spine boundary, and (3) detecting vertebrae. Details are described as follows.

**Figure 7:**
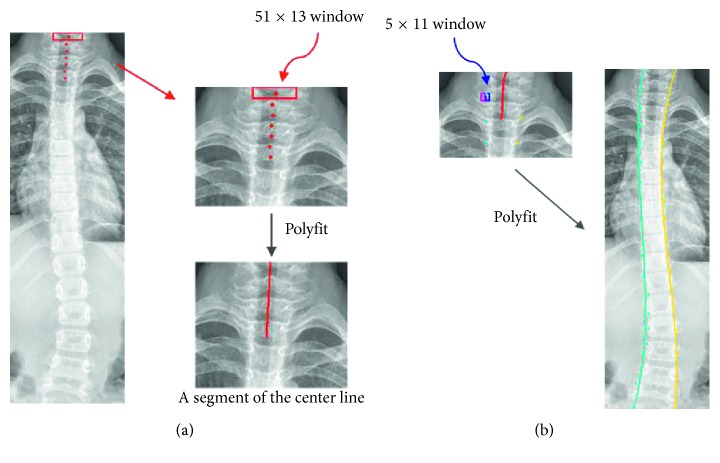
Finding boundary points of the spine: (a) finding a segment of the center line before finding spinal edges; (b) using a segment of center line for finding spinal edges.

The first step of vertebrae detection is to detect the central line segment (CLS) of the vertebrae. In this step, many rectangle windows with a size of *H* × *W* pixels are overlapped and placed with one-pixel increment along the top of the spine ROI from left to right. The sums of intensity inside each rectangle window are calculated. If one rectangle window has the largest sum of intensity, the top middle point of this window is used as the first reference point for CLS as shown in Figure 7(a). Next, the current rectangle window with a maximum sum of intensity is moved down *p* pixels, and then a search is initiated for the next reference point in the interval of *q* pixels on both of its sides. This search slips one pixel once and then records the intensity sum of the corresponding window. The window with the maximum sum of intensity value is then assigned to the current window and its top middle point is defined as the second reference point for CLS. Similar procedures are repeated until *n* reference points are detected, and these are then fit into a CLS by polynomial fitting method as shown in Figure 7(a).

The boundary points of the spine along the normal direction of the detected central line segment are determined in the second step. This second step utilizes two small sibling windows, each 11 × 5 pixels. The pair of sibling windows moves at most *r* pixels along both sides in normal directions of the corresponding CLS point as shown in Figure 7(b). The top middle of the pair of the sibling windows is selected as the boundary point of the spine when their intensity difference is maximum as shown in Figure 7(b). The procedure for boundary detection continues until all points of the CLS have been explored. The corresponding current window of the final point for this CLS is reconstructed for sequential detection of the CLS until all boundaries of the spine are found. Finally, all spine boundary points in each side are dependently fitted by polynomial fitting with three degrees into the spine boundary. In the experiments, we set the following parameters: *H* = 51, *W* = 13, *p* = 12, *q* = 10, *r* = 40, and *n* = 6.

**Figure 8:**
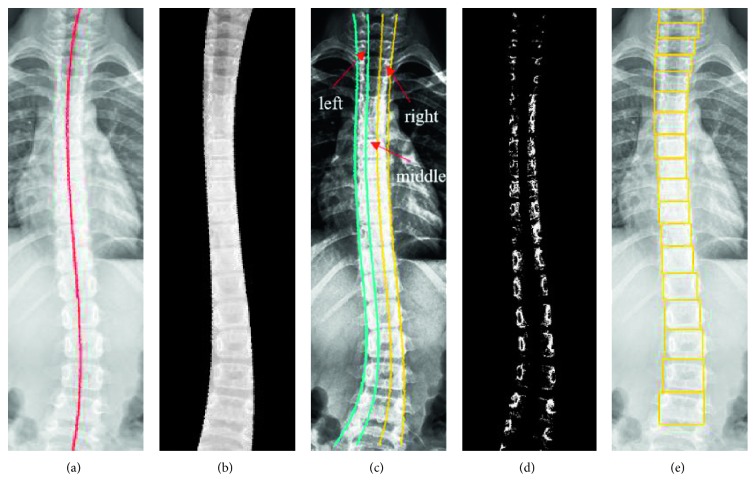
Detection of vertebrae: (a) central spinal curve line, (b) spine foreground, (c) region partition, (d) threshold result, and (e) results of vertebra detection.

Once the right and left boundaries of the spine are obtained, we consider the middle point of the pair of the boundary in the horizontal line as a point of the central spinal curve (CSC) line. The complete CSC line and region for the foreground of spine are drawn in Figures 8(a) and 8(b). The results are then applied in the final procedure for vertebrae detection. The spinal area enclosed by the two boundary lines is equally divided into three regions: left, middle, and right, as shown in Figure 8(c). The left and right regions are used to generate threshold images *I*_*t*_ with threshold values *γ*_*t*_=16*t*, *t*=1,…, 15.

Figure 8(d) shows image *I*_12_ in which the region of the vertebrae always appears in the brightest region. The intensity of each *I*_*t*_ image is projected normally to the CSC line and then summed up in their projection histogram *p*_*t*_. The transformed projection *f*_*t*_ is generated by the following equation:

(1) $$\begin{array}{c} f_{t}\left(y\right)=\left\{\begin{array}{cc} 0, & \text{if}\;\;p_{t}\left(y\right)>0,\\ 1, & \text{otherwise,} \end{array}\right. \end{array}$$

where *y* is an index of the histogram, that is *y*=1,…, *β*, where *β* is the bin dimension of histogram *p*_*t*_. In general, *β* is the length of the spine's central line. The accumulated histogram *P* is the sum of all *f*_*t*_ shown as follows:

(2) $$\begin{array}{c} P\left(y\right)=\overset{15}{\underset{t=1}{\sum }}f_{t}\left(y\right). \end{array}$$

The computation for histogram P looks like a voting mechanism; more precisely, the pixels of the intervertebral disc region always have a larger value than those of a vertebra. The value of the histogram in the vertebrae is almost always assigned to be 0. In order to obtain the rectangle region of interest (ROI) for a vertebra, we first select each drastic change in the increasing order of histogram P as starting point A. In general, the start point always occurs in the lower bound of each vertebra, i.e., the border point between vertebra and the lower intervertebral disc. Starting from each point A along the CSC lines, we extract a 15 bin nonoverlapped subhistogram from the corresponding P histogram. The first finding of a global maximum of each subhistogram indicates as the position of the horizontal boundary line of the ROI of the corresponding vertebrae. The ROI of vertebrae enclosed by two adjacent horizontal lines and the spine boundary are defined as the region of interested of the vertebrae as shown in Figure 8(d).

#### 2.2.3. Segmentation of Vertebrae

After the vertebrae detection step, we obtain the 17 vertebrae regions of interest (ROI) of each spine image. In the AP view spine images, the vertebrae intensity varies considerably, but in general, cervical vertebrae usually have a low intensity and the lumbar vertebrae usually appear with a very high intensity. Intensity inconsistency makes it difficult to segment by using only simple image processing techniques. Thus, current convolutional neural network (CNN) techniques have become a powerful alternative to overcome the problem of intensity inconsistence. Essentially, CNN is an end-to-end mechanism where the inputs to the CNN are the original images without applying any image processing procedure. All vertebra regions of interest are rescaled as input images with a size of 256 × 128 pixels for the CNN segmentation. We then applied three different convolutional neural networks (CNN) : U-Net, Residual U-Net, and Dense U-Net, to segment vertebrae and for comparison.

**Figure 9:**
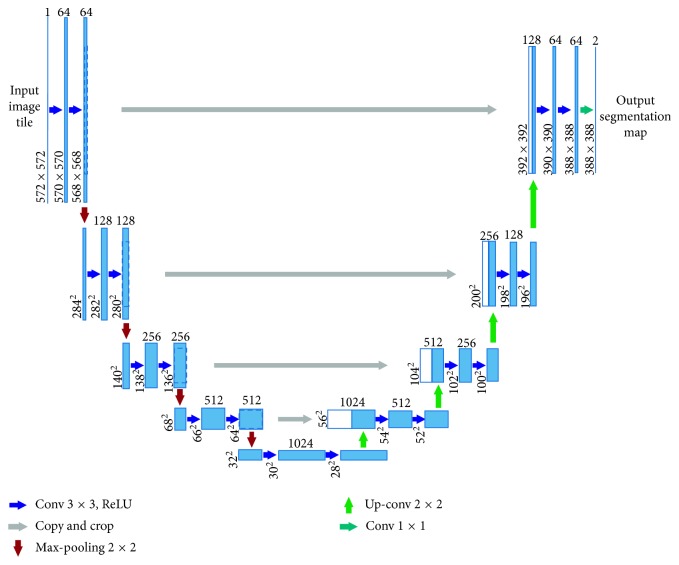
The original architecture of U-Net [19].

U-Net is based on an encoder-decoder structure, which was originally developed and used for the biomedical image segmentation [19] as shown in Figure 9.

**Figure 10:**
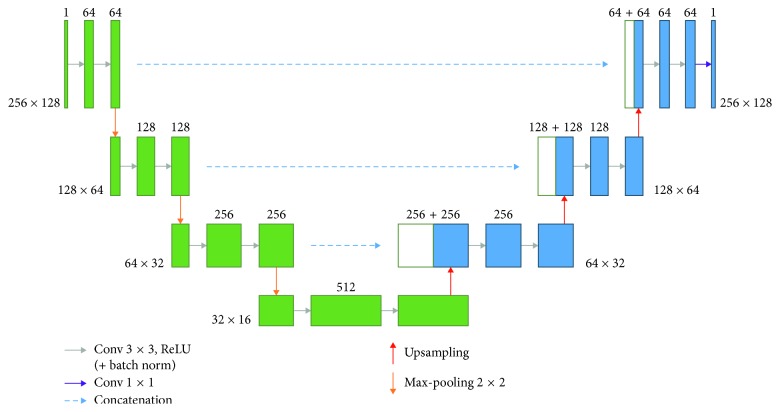
U-Net architecture of the proposed method.

We revised the original U-Net architecture to be suitable for the vertebra segmentation as shown in Figure 10. The left side of the proposed U-Net is the encoder part and the right side is the decoder part. The encoder part applies convolution and downsampling to extract information into feature maps from the input image. The decoder part reconstructs the prediction map from the encoded feature maps by using upsampling and concatenation of corresponding feature maps from the encoder side. In the original U-Net, the crop and copy operation needs to crop the central area of feature map of encoder part and then concatenate them with the corresponding feature map in the decoder stage. However, the crop operation always loses important information of vertebra segmentation. In order to avoid important information loss, we replace the original crop and copy operation with concatenation operation in the design of U-Nets. Similar strategy has also been adopted in the other literatures [20]. A vertebra ROI image with a size of 256 × 128 pixels was input to the network for segmentation.

In the convolutional layers, the operation with a 3 × 3 filter convolution was performed, followed by a rectified linear unit (ReLU) [26] and batch normalization (BN) [27] that was applied in both the encoder and decoder part of the network. The convolution is applied by learnable filters to extract features from the input image.

In our network, the convolution of an image is performed by filters with size 3 × 3, stride 1 to generate feature maps. The equation of convolution is denoted as follows:

(3) $$\begin{array}{c} h\left(x_{l}\right)=W^{T}\;*\;x_{l}+b, \end{array}$$

where *x*_*l*_ and *h*(*x*_*l*_) are input and output in the *l*^th^ layer of the convolution, respectively, *W*^*T*^ is a learning filter of the convolution, and *b* is a bias.

A rectified linear unit (ReLU) [26] is a kind of activation function and is applied for nonlinear transformation for the feature maps. ReLU is commonly used because it has lower computational costs and better performance than other activation functions in typical cases. The ReLU activation function is expressed as below:

(4) $$\begin{array}{c} ℱ\left(x_{l},w_{l}\right)=f\left(h\left(x_{l}\right)\right)=\max\left(0,h\left(x_{l}\right)\right), \end{array}$$

where *f*(·) is the activation function and *ℱ*(*x*_*l*_, *w*_*l*_) represents the output of the *l*^th^ convolution layer under the *w*_*l*_ weight. In the network, the output feature maps are downsampled or upsampled after two convolutional layers.

The 2 × 2 max-pooling operation with stride 2 is applied for downsampling in the encoder part. The purpose of pooling operation is downsampling, which is used to reduce the size of the feature maps. In this study, we use max-pooling which outputs the maximum value within the window regions. Max-pooling can make learned features more robust and reduce noise. The decoder part resizes the feature map by using deconvolution at upsampling, followed by a 3 × 3 filter size convolution that halves the number of feature channels, and the output concatenates with the corresponding feature map from the encoder part. At the final layer, a 1 × 1 filter convolution is applied to map the 64 channels of a feature map to a probability map in the range of [0, 1], and the segmentation result is generated after probability thresholding.

**Figure 11:**
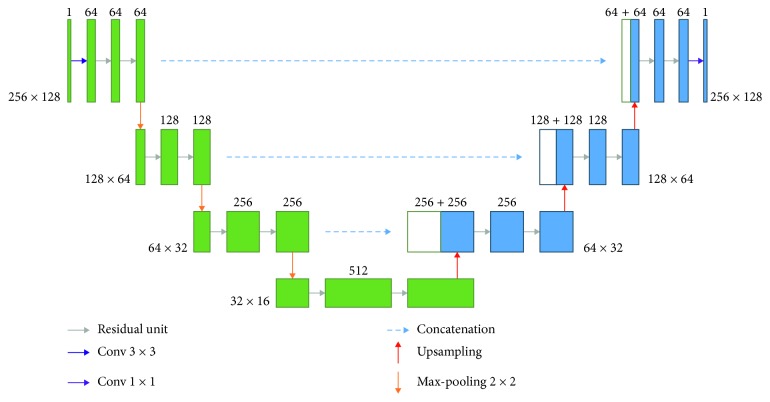
Residual U-Net architecture of the proposed method.

Our next proposed network architecture based on a Residual U-Net [24] is shown in Figure 11. The architecture of the Residual U-Net is similar to that of U-Net as mentioned previously.

**Figure 12:**
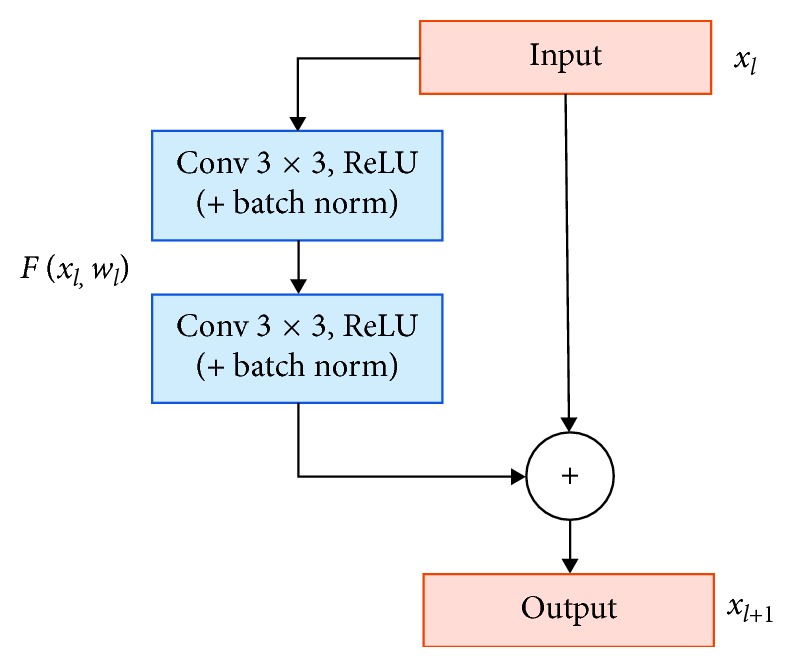
Residual block [28].

The difference between U-Net and Residual U-Net is that Residual U-Net replaces the standard convolution operation of U-Net with a residual block. The concept of a residual block which is applied on network is proposed by He et al. [28]. In their research, the proposed network, named a residual neural network, was used to improve the performance of the network and address the degradation problem. As shown in Figure 12, each residual block contains two repeated operations which included batch normalization, ReLU and 3 × 3 filter convolution, and identity mapping. The identity mapping connects input to the output of the block. Each residual block can be calculated as follows:

(5) $$\begin{array}{c} x_{l+1}=x_{l}+ℱ\left(x_{l},w_{l,k}\right), \end{array}$$

where *x*_*l*_ and *x*_*l*+1_ are the input and output of the *l*-th residual block, respectively, *w*_*l*,*k*_ is the weight of the first residual unit, and *k* is the number of weighted layers contained in each residual unit. The *ℱ*(·) is the residual function stacking two 3 ∗ 3 convolutional layers.

**Figure 13:**
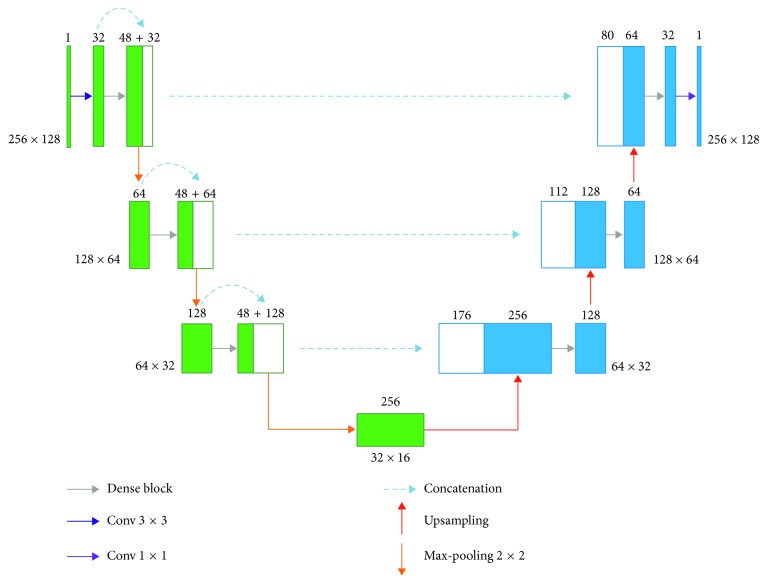
Dense U-Net architecture.

A Dense U-Net [25] is the architecture of a U-Net built from dense blocks [29]. The architecture of a Dense U-Net is shown in Figure 13.

**Figure 14:**
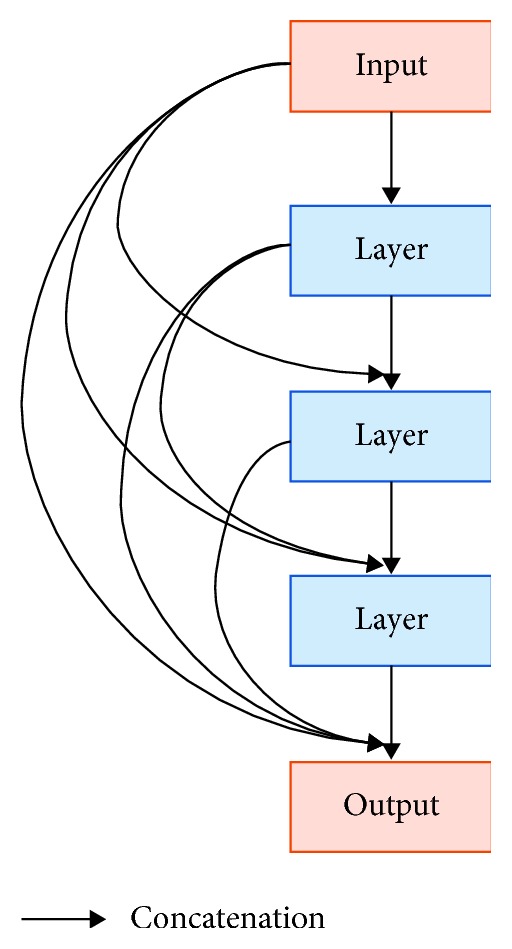
Dense block [29].

As known from the Residual U-Net above, the input is added to the output of a layer in a residual block. In a dense block, all feature layers are connected and then have concatenation applied instead of addition. Each dense block can be calculated as follows:

(6) $$\begin{array}{c} x_{l}=H_{l}\left(\left[x_{0},x_{1},\ldots ,x_{l-1}\right]\right), \end{array}$$

where [*x*_0_, *x*_1_,…, *x*_*l*−1_] indicates the concatenation of the feature maps produced in layers 0, … , l − 1. *H*(·) is a dense layer that includes batch normalization, rectified linear units (ReLU), and convolution layer. A *l*-layer dense block with a growth rate of *k* outputs *l* × *k* feature maps, as shown in Figure 14.

**Figure 15:**
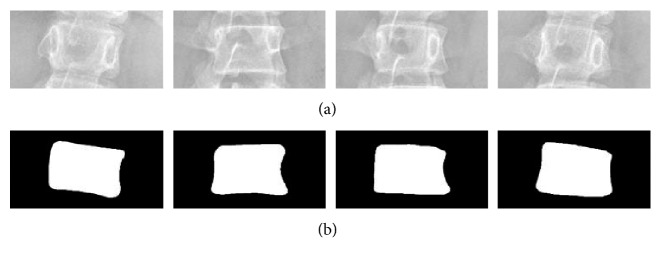
Vertebra images: (a) the ROI of vertebra and (b) their corresponding annotated segmentation ground truth.

In our implementations, the dataset consisted of 595 vertebra images. The boundary of each vertebra image was annotated by clinical experts.  Figure 15 shows the vertebra images and their corresponding segmentation ground truth. The 5-fold cross-validation was used to evaluate the segmentation performances of U-Net, Residual U-Net, and Dense U-Net. In each fold, the training images were augmented to 1000 images, 10% of them were used as the validation images.

All parameters of the CNN network are randomly initialized and trained by an Adam optimizer. The loss function for the network optimization uses the L2-norm loss function by minimizing of the sum of the square of the differences between the predicted result and the ground truth. The loss function is calculated by

(7) $$\begin{array}{c} \text{L}2\text{ loss}=\frac{\sum_{i=0}^{N}{\left(y_{i}-h\left(x_{i}\right)\right)}^{2}}{N}, \end{array}$$

where *x*_*i*_ is input data, *y*_*i*_ is ground truth, *h*(*x*_*i*_) is the predicted result, and *N* is number of data.

### 2.3. Cobb Angle Measurement

**Figure 16:**
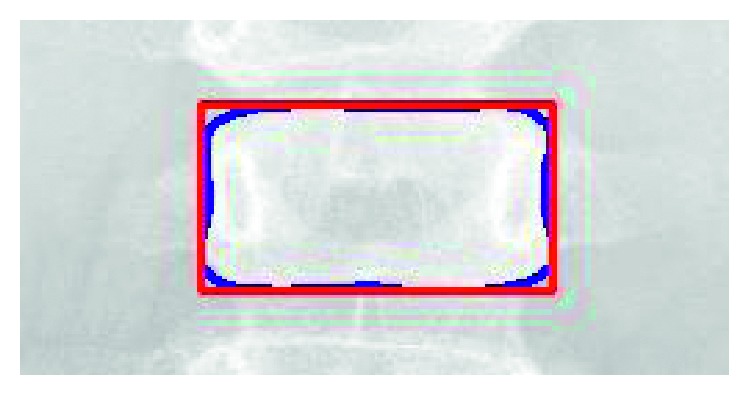
The minimum bounding rectangle (MBR) approach for obtaining the upper and lower borders of the vertebra.

Cobb angle [3] is the most widely used measurement for quantifying spine curvature. The curvature of the Cobb method is defined as the angle between the upper border of the upper vertebra and the lower borders of the lowest vertebra as shown in Figure 3. The definition of the upper and the lower border in the original approach is determined by manual drawing lines parallel to the upper and lower borders to find the angle. In our implementations, we used an automatic approach called minimum bounding rectangle (MBR) method to obtain the upper and lower border of the vertebra. For the MBR method, we find a minimum bounding rectangle according to the segmented vertebral contour and then consider the top and bottom border of this rectangle as the upper and lower borders of vertebra.  Figure 16 shows an example for MBR approach.

Once the upper and lower borders are defined, we can calculate spine curvature *φ* by the formula below:

(8) $$\begin{array}{c} \varphi =\max\left\{\left|\tan^{-1}\left(\frac{m_{i}-m_{j}}{1+m_{i}\times m_{j}}\right)\right|\right\},\;\left(i,j\right)\in \left\{\left(a,b\right)\left|a\in N\right.,b\in N,b-a\geq 2\text{ and }b\leq N\right\}, \end{array}$$

where *a* is the upper vertebra and *b* is the lower vertebra with at least one vertebrae interval from upper vertebra *a*. *m*_*i*_ is the slope of the upper border of the upper vertebra and *m*_*j*_ is the slope of the lower border of the lower vertebra. *N* is the number of counted vertebrae. We calculated all possible curvatures of the spine and consider the maximum curvature as the resulting Cobb angle.

## 3. Experimental Results and Discussions

The experiments were performed on a PC with Intel Core i7 3.60 GHz CPU, 16 GB memory, and NVIDIA GeForce GTX 1080Ti GPU. The network was implemented based on the Tensorflow framework in Python. In this section, we evaluated the performance metrics of the proposed system. There are six performance metrics, including accuracy (AC), sensitivity (SE), specificity (SP), mean square error (MSE), Dice similarity coefficient (DSC) [30], and Jaccard similarity (JS) [31], which were used for quantitative analysis of the experimental results and are defined below:

(9) $$\begin{array}{c} \text{accuarcy}\left(\text{AC}\right)=\frac{\text{TP}+\text{TN}}{\text{TP}+\text{TN}+\text{FP}+\text{FN}},\\ \text{sensitivity}\left(\text{SE}\right)=\frac{\text{TP}}{\text{TP}+\text{FN}},\\ \text{specificity}\left(\text{SP}\right)=\frac{\text{TN}}{\text{TN}+\text{FP}},\\ \text{dice similarity coefficient}\left(\text{DSC}\right)=\frac{2\left|\text{GT}\cap \text{SR}\right|}{\left|\text{GT}\right|+\left|\text{SR}\right|},\\ \text{Jaccard similarity}\left(\text{JS}\right)=\frac{\left|\text{GT}\cap \text{SR}\right|}{\left|\text{GT }\cup \text{ SR}\right|},\\ \text{mean square error}\left(\text{MSE}\right)=\frac{1}{N}\overset{n}{\underset{i=1}{\sum }}{\left({\text{GT}}_{i}-{\text{SR}}_{i}\right)}^{2}, \end{array}$$

where GT is the ground truth, SR is the segmentation result, and *N* is the number of all images.

### 3.1. Evaluation of U-Net, Residual U-Net, and Dense U-Net

In the experiments, we evaluated the segmentation performance of the U-Net, the Residual U-Net, and Dense U-Net, where they were trained according to the following parameters: batch size is 10, learning rate is 0.01, and the number of epoch until stopping is 100.

**Figure 17:**
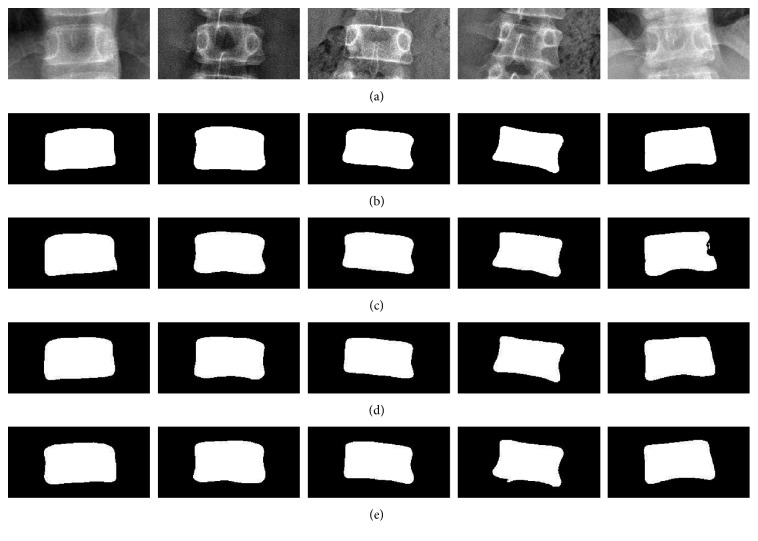
Segmentation results of vertebrae: (a) the input image, (b) ground truth, (c) segmented result of U-Net, and segmented results of (d) Residual U-Net and (e) Dense U-Net.

Table 2 shows the Dice similarity coefficient (DSC) from a 5-fold cross-validation of U-Net, Residual U-Net, and Dense U-Net and their usage for parameter size, training time, and test time of each image. From Table 2, the DSC performance of Residual U-Net is 0.951 which is better than that of U-Net and Dense U-Net. The result is also superior to the results in [20]. The segmented results of the U-Net, the Residual U-Net, and Dense U-Net are shown in Figure 17. In Figure 17, the first row shows the input original images, the second row the ground truth, the third row is the segmentation results for U-Net, the fourth row is the results for Residual U-Net, and the last row is the results of Dense U-Net. The segmentation results of the three networks are highly fitted to the ground truth on the left two cases. However, there are some artifacts and less segmentation on the results of U-Net for the remaining cases, although the results of Residual U-Net are still pretty good. This shows that our proposed Residual U-Net is promising for vertebra segmentation.

**Figure 18:**
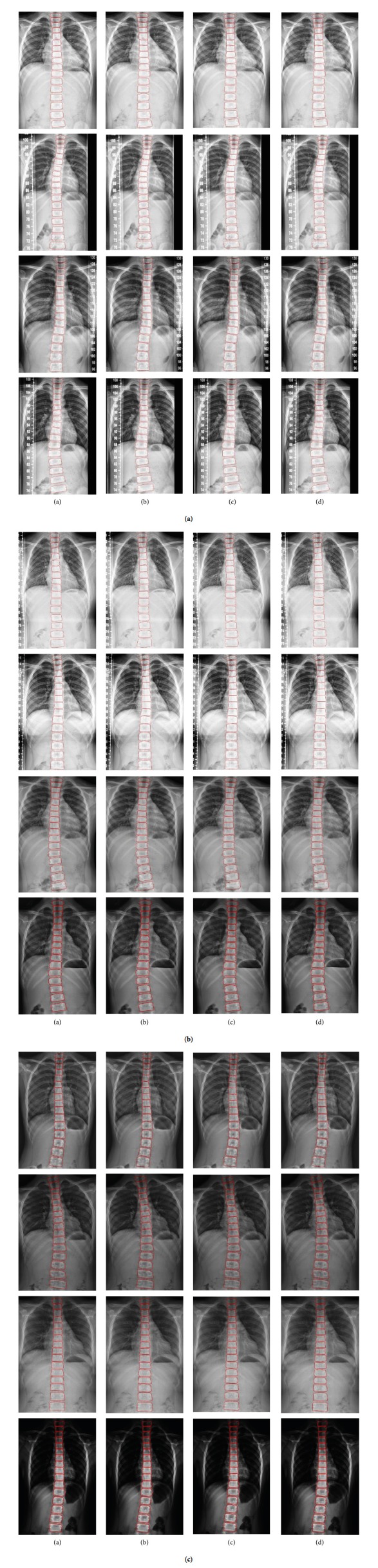
Segmentation results of a whole spine: the ground truth (a); the results of U-Net, Residual U-Net, and Dense U-Net are shown in (b), (c), and (d), respectively.

Additionally, we also applied several performance metrics to quantitatively evaluate the segmented results for the U-Net and Residual U-Net. The DSC, JS, MSE, accuracy, sensitivity, and specificity are shown in Table 3. The Residual U-Net had the best performance for all these metrics compared to U-Net and Dense U-Net. After vertebrae segmentation, the results are drawn in the original spine image to show the spine segmentation results as depicted in Figure 18. Figure 18 shows the vertebrae segmentation results for U-Net, Residual U-Net, and Dense U-Net, in which the first column is the ground truth, the second to fourth columns show the results for U-Net, Residual U-Net, and Dense U-Net, respectively. The upper and lower boundaries of vertebrae are proper and better fit to the ground truth for the Residual U-Net results and are close to those of Dense U-Net. This demonstrates that the performances of both the Residual U-Net and Dense U-Net methods are suitable for spine curvature estimation tasks. From clinical experts' perspectives, the proposed vertebrae segmentation does not need the manual intervention which is time consuming and instable. The proposed method provides rapid response and precise measurement.

### 3.2. Evaluation of Spine Curvature Result and Ground Truth

In this experiment, we compared the results of the Cobb method with manual results that were measured by two orthopedists (one is an expert, the other is a novice) as shown in Table 4. Each orthopedist measures the same X-ray spinal images twice in different times. The results of this table indicate that the spine curves to right when the angle of spine is less than 0, and the spine curves to left when the angle is larger than 0.

Statistical analysis was performed using the software toolkit designed by Jason Brownlee [32]. The descriptive statistic includes mean, standard deviation, and 95% confidence interval, which were used to explain the findings of the experimental results. The purpose of a one-way analysis of variance (one-way ANOVA) [33] is to compare the means of two or more groups (the independent variable) on one dependent variable to see if the group means are significantly different from each other. Thus, the one-way analysis of variables was used to analyze the difference among the three measurement results while also considering their signs. The one-way ANOVA analysis, with its corresponding statistics = 0.020 and *p*=0.980 was less than the significant level *α* = 0.05. This result fails to reject the null hypothesis such that the three data samples have the same distribution, i.e., no significant difference.

Furthermore, the reliability of the Cobb angle measured by using our proposed MBR method was assessed by the intraclass correlation coefficient (ICC) [34, 35, 36, 37] and Pearson correlation coefficient [38]. In general, the ICC values are rated poor (less than 0.40), fair (0.40–0.59), good (0.0–0.74), or excellent (0.75–1.00). The levels of significance in the experiments were set at *p* < 0.05. The experimental results of intraclass and interclass correlation coefficients are shown in Table 5. The ICC and Pearson correlation coefficient were more than 0.93, indicating the results of MBR were highly matched with the manual assessment.

Rank correlation can be calculated for real-valued variables. This is done by first converting the values for each variable into rank data. This is where the values are ordered and assigned an integer rank value. Rank correlation coefficients can then be calculated in order to quantify the association between the two ranked variables. Because no distribution is assumed for the values, rank correlation methods are referred to as a distribution-free correlation or nonparametric correlation. Interestingly, rank correlation measures are often used as the basis for other statistical hypothesis tests, such as determining whether two samples were likely drawn from the same (or different) population distributions.

An analysis of the severity ranks applied the Spearman rank-order correlation [39]. This is also called Spearman's correlation coefficient and is denoted by the lowercase Greek letter rho (*ρ*). As such, it may be referred to as Spearman's rho. This statistical method quantifies the degree to which ranked variables are associated by a monotonic function, reflecting an increasing or decreasing relationship. As a statistical hypothesis test, the method assumes that the samples are uncorrelated (fail to reject H0).

(10) $$\begin{array}{c} \rho =1-\frac{6\sum_{i=1}^{n}{\left(x_{i}-y_{i}\right)}^{2}}{n\left(n^{2}-1\right)}, \end{array}$$

where *x*_*i*_ and *y*_*i*_ are the rank of *i*th sample of different data samples and *n* is the number of samples.

The results for different pairs of data samples for Cobb measurement are shown in Table 6, where the results “rejected the null hypothesis.” This means that the three ranked data samples were highly correlated. The highly correlated result indicates that the Cobb measurement obtained by the MBR method has high potential as a new indicator for diagnosing the severity of scoliosis. In addition, the averaged differences of measurements when different physicians manually computed twice were 1.93° and 0.21°. Apparently, the manual computation usually suffers with intraclass error measurement.

## 4. Conclusion and Future Work

In this study, we proposed an automatic measurement system for assessing the severity of scoliosis. The system consists of three main parts: isolation of the spine, vertebra segmentation, and Cobb angle measurement. In the segmentation of the vertebra, we applied and compared three different convolutional neural networks (CNN) that are the original U-Net, Residual U-Net, and Dense U-Net. The segmentation results of Residual U-Net were superior to the other two methods. Its average Dice similarity coefficient reached up to 0.951. The one-way ANOVA analysis of our proposed MBR measurement of Cobb angle and results by manual computation by two clinical doctors indicated that the results do not have any significant differences. The test of Spearman rank-order correlation showed that the MBR results of our proposed method were highly correlated to manual assessment by clinical doctors.

The main contribution of this study is a method that provides a reliable and convenient measurement of the Cobb angle for clinical applications. MBR measurement only focuses on the computation of Cobb angle for spine curvature. Other interesting features such as the length of central spinal curve (CSC) and the ratio of curvature to CSC are also proven effective measurements for assessing the severity of scoliosis [34]. In addition, many novel studies now build a three-dimensional model of the spinal volume to assist in more accurate detection and measurement of spine curvature [18, 40]. As such, current and future studies are exploring various promising methods to develop more accurate measurement of the Cobb angle of the spine to assess scoliosis.

## Figures and Tables

**Table 1:** Definition of Cobb angle [5].

Cobb angle	Definition
0°–10°	Spinal curve
10°–20°	Mild scoliosis
20°–40°	Moderate scoliosis
>40°	Severe scoliosis

**Table 2:** Dice similarity coefficient (DSC) from 5-fold cross-validation of U-Net, Residual U-Net, and Dense U-Net.

*k*-fold	Dice similarity coefficient (DSC)		
	U-Net	Residual U-Net	Dense U-Net
*k* = 1	0.940 ± 0.036	0.952 ± 0.023	0.947 ± 0.028
*k* = 2	0.942 ± 0.032	0.951 ± 0.029	0.947 ± 0.029
*k* = 3	0.942 ± 0.033	0.952 ± 0.025	0.949 ± 0.028
*k* = 4	0.941 ± 0.034	0.949 ± 0.030	0.947 ± 0.026
*k* = 5	0.942 ± 0.035	0.952 ± 0.028	0.947 ± 0.030
Average ± std.	0.941 ± 0.034	0.951 ± 0.027	0.948 ± 0.028
Parameter size	1.21 million	1.19 million	1.20 million
Training time	0.34 hour	0.77 hour	2.33 hour
Testing time (each image)	0.03 second	0.05 second	0.07 second

**Table 3:** Quantitative evaluation of the segmented results of U-Net, Residual U-Net, and Dense U-Net.

Methods	Dice similarity coefficient (DSC)	Jaccard similarity coefficient (JS)	Mean square error (MSE)	Accuracy (AC)	Sensitivity (SE)	Specificity (SP)
U-Net	0.941 ± 0.034	0.891 ± 0.057	0.030 ± 0.016	0.961 ± 0.022	0.980 ± 0.016	0.945 ± 0.038
Residual U-Net	0.951 ± 0.027	0.908 ± 0.046	0.025 ± 0.012	0.969 ± 0.016	0.982 ± 0.013	0.958 ± 0.029
Dense U-Net	0.948 ± 0.028	0.902 ± 0.048	0.027 ± 0.013	0.966 ± 0.017	0.982 ± 0.014	0.952 ± 0.031

**Table 4:** Results of spine curvature with Cobb method and manual method.

Image	Observer 1 (expert)						Observer 2 (novice)						Cobb method (MBR)		
	Upper vertebrae		Lower vertebrae		Cobb angle		Upper vertebrae		Lower vertebrae		Cobb angle		Upper vertebrae	Lower vertebrae	Cobb angle
	*t* = 1	*t* = 2	*t* = 1	*t* = 2	*t* = 1	*t* = 2	*t* = 1	*t* = 2	*t* = 1	*t* = 2	*t* = 1	*t* = 2			
1	T8	T8	L2	L2	−16.8	−16.9	T8	T8	L1	L1	−15.0	−15.2	T8	L5	−20.1
2	T6	T9	L1	L1	6.4	6.1	T10	T10	L1	L1	13.7	13.7	T6	T12	7.8
3	T2	T10	L1	L2	9.9	6.2	T4	T4	L2	L2	11.6	11.6	T3	L2	10.1
4	T9	T10	L4	L4	11.9	16.9	T9	T9	L4	L4	13.9	13.9	T11	L4	15.9
5	T11	T10	L4	L4	15.9	14.5	T11	T11	L4	L4	11.6	11.6	T12	L4	9.1
6	T11	T9	L4	L4	−19.2	−16.8	T10	T10	L4	L4	−15.1	−15.1	T11	L3	−15.1
7	T12	T11	L4	L4	−8.1	−12.3	T9	T9	L4	L4	−12.0	−12.0	T6	L2	−5.2
8	T12	T11	L4	L4	−9.1	−8.2	T9	T9	L4	L4	−13.5	−13.5	T12	L2	−11.0
9	T10	T10	L4	L4	−19.8	−15.6	T11	T11	L4	L4	−20.6	−20.6	T9	L3	−14.8
10	T11	T12	L4	L4	10.2	11.0	T12	T12	L4	L4	10.9	10.9	T12	L3	10.8
11	T5	−	L1	−	−8.4	0	T7	T7	L2	L2	−4.2	−4.2	T1	T12	−7.2
12	T5	T5	L2	L1	13.5	8.7	T4	T4	L3	L3	9.3	9.3	T1	L2	11.1
13	T10	T10	L4	L4	15.1	14.0	T10	T10	L4	L4	14.4	14.4	T9	L5	13.5
14	T9	T9	L4	L4	−15.4	−12.1	T10	T10	L4	L4	−13.8	−13.8	T2	L5	−14.2
15	No scoliosis						T4	T4	T12	T12	−7.4	−7.4	T4	T10	−7.7
16	T9	T11	L4	L4	−14.2	−15.0	T7	T7	L4	L4	−20.2	−20.2	T7	L4	−18.9
17	T1	T4	T7	T12	5.9	8.4	T2	T2	T12	T12	13.4	13.6	T2	T12	11.3
18	T7	T6	L1	L3	14.7	8.3	T6	T6	L4	L4	7.0	7.1	T9	L1	7.6
19	T11	T9	L5	L5	−6.9	−9.6	T4	T4	L5	L5	−11.5	−11.7	T2	L5	−12.4
20	T3	T2	T8	T11	7.1	8.5	T4	T4	T6	T6	8.8	8.5	T3	T6	9.8
21	T12	T10	L5	L4	11.1	9.9	C3	C3	L2	L2	15.3	16.2	T3	L3	13.8
22	T7	T7	L4	L4	12.9	13.0	T2	T2	L4	L4	16.8	16.9	T3	L3	18.1
23	T8	T8	L3	L4	11.3	13.8	T11	T11	L5	L5	15.5	15.6	T11	L3	9.4
24	T7	T9	L4	L4	−14.1	−14.0	T5	T5	L5	L5	−21.8	−22.1	T2	L3	−13.5
25	T9	T8	L3	L3	−16.2	−14.5	T6	T6	L3	L3	−10.3	−9.9	T11	L2	−10.7
26	T8	T8	L3	L3	−8.2	−8.0	T7	T7	L2	L2	−6.4	−6.3	T2	L2	−5.6
27	T5	T5	L3	L4	−17.3	−17.3	T5	T5	L3	L3	−15.8	−15.0	T1	L3	−15.2
28	T11	T11	L4	L3	18.4	15.7	T12	T12	L4	L4	22.4	23.1	T12	L4	16.6
29	No scoliosis						T3	T3	T10	T10	−5.2	−5.1	T2	L4	−6.6
30	T9	T9	L4	L4	−11.7	−9.9	T5	T5	L4	L4	−14.2	−14.1	T6	L4	−8.7
31	T11	T12	L4	L4	−6.9	−7.7	T3	T3	L1	L1	10.0	10.1	L1	L4	−7.4
32	T11	T10	L4	L4	−5.9	−6.0	T5	T5	L5	L5	−6.2	−7.1	T1	L3	−9.1
33	T9	T9	L4	L4	−16.7	−16.0	T8	T8	L4	L4	−14.0	−13.3	T11	L4	−12.5
34	T12	T12	L4	L4	10.3	12.1	T12	T12	L4	L4	10.0	9.7	T1	L3	9.9
35	T5	T7	L1	L2	15.7	16.4	T5	T5	L1	L1	17.0	17.9	T5	L1	13.8

*Note.* The “no scoliosis” or empty data indicate that the result of manually measurement is no scoliosis. Their Cobb angle is assigned to be 0 in statistical analysis. Each orthopedist measures the same X-ray spinal images twice in different times (*t* = 1 or *t* = 2).

**Table 5:** The statistical data of a MBR Cobb measurement.

Variable	Observer (expert)	Observer (novice)	MBR (proposed method)
Cobb's angle	−0.703 ± 12.552 (−19.8 to 18.4)	−0.106 ± 13.582(−21.8 to 22.4)	−0.694 ± 12.091(−20.1 to 18.1)
Intrareliability (ICC)	0.936 (expert-novice)	0.9710 (expert-MBR)	0.940 (novice-MBR)
Pearson correlation coefficient	0.944 (expert-novice)	0.971 (expert-MBR)	0.948 (novice-MBR)

Data are presented as mean ± standard deviation with 95% confidence interval and compared using the one-way analysis of variance (ANOVA).

**Table 6:** The Spearman rank-order correlation of different data pair of Cobb measurement.

Data pair of Cobb measurement	Expert-novice pair	Expert-MBR pair	Novice-MBR pair
Spearman rank-order correlation (*α* < 0.05)	0.889, *p*=0.000 (reject H0)	0.891, *p*=0.000 (reject H0)	0.928, *p*=0.000 (reject H0)

## Data Availability

The data used to support the findings of this study are available from the corresponding author upon request.
